# Facile Synthesis of Mesoporous Nanohybrid Two-Dimensional Layered Ni-Cr-S and Reduced Graphene Oxide for High-Performance Hybrid Supercapacitors

**DOI:** 10.3390/ma16196598

**Published:** 2023-10-08

**Authors:** Ravindra N. Bulakhe, Anh Phan Nguyen, Changyoung Ryu, Ji Man Kim, Jung Bin In

**Affiliations:** 1 Soft Energy Systems and Laser Applications Laboratory, School of Mechanical Engineering, Chung-Ang University, Seoul 06974, Republic of Korea; bulakhern@gmail.com (R.N.B.); asd24zx@cau.ac.kr (C.R.); 2 Department of Chemistry, Sungkyunkwan University, Suwon 16419, Republic of Korea; jimankim@skku.edu; 3 Department of Intelligent Energy and Industry, Chung-Ang University, Seoul 06974, Republic of Korea; anhpn@cau.ac.kr

**Keywords:** electrochemical impedance spectroscopy, energy density, hybrid supercapacitor, nickel-chromium-sulfide (Ni-Cr-S), specific surface area

## Abstract

This study describes the single-step synthesis of a mesoporous layered nickel-chromium-sulfide (NCS) and its hybridization with single-layered graphene oxide (GO) using a facile, inexpensive chemical method. The conductive GO plays a critical role in improving the physicochemical and electrochemical properties of hybridized NCS/reduced GO (NCSG) materials. The optimized mesoporous nanohybrid NCSG is obtained when hybridized with 20% GO, and this material exhibits a very high specific surface area of 685.84 m^2^/g compared to 149.37 m^2^/g for bare NCS, and the pore diameters are 15.81 and 13.85 nm, respectively. The three-fold superior specific capacity of this optimal NCSG (1932 C/g) is demonstrated over NCS (676 C/g) at a current density of 2 A/g. A fabricated hybrid supercapacitor (HSC) reveals a maximum specific capacity of 224 C/g at a 5 A/g current density. The HSC reached an outstanding energy density of 105 Wh/kg with a maximum power density of 11,250 W/kg. A 4% decrement was observed during the cyclic stability study of the HSC over 5000 successive charge–discharge cycles at a 10 A/g current density. These results suggest that the prepared nanohybrid NCSG is an excellent cathode material for gaining a high energy density in an HSC.

## 1. Introduction

Supercapacitors (SCs) are classified into three types: electric double-layer capacitors (EDLCs), pseudocapacitors, and hybrid SCs (HSCs) [1,2]. The EDLC [3] has limited specific energy due to a simple electrostatic charge storage mechanism and pseudocapacitor [4]. The charge transfer is limited at the electrode surface and electrolyte interface, compromising SCs’ capability of ultra-high power and a long cycle life [2,5]. Therefore, the combination of high specific power and energy and long cycle life in one cell has been a research goal for energy storage devices. In addition, HSCs are one of the best alternative solutions to increasing specific energy and maintaining high specific power without sacrificing the cyclic stability of SC devices [5,6,7]. The HSC integrates the advantages of the EDLC and pseudocapacitor, minimizing their disadvantages, and is classified into three categories: asymmetric, composite, and battery-type, according to the nature of their anode and cathode materials.

Among them, battery-type hybrid materials display the best supercapacitive performance, offering a high charge storage capacity, operating voltage, and capacity retention; a long lifetime; and outstanding specific energy and power due to their unique charge storage mechanism of deep and diffusion-limited intercalation [8,9,10]. The charge storage mechanism of SCs depends on the composition and physicochemical properties of the electrode materials (e.g., conductivity, porosity, wettability, specific surface area, redox properties, etc.). Therefore, the charge stored in the SC is directly proportional to multiple faradic and non-faradic reactions occurring at a) the interface of the electrode surface with electrolytes and b) the interplanar surface of the layered materials [11,12].

Many researchers have used various materials and methods, such as carbon materials and their derivatives [13], metal hydroxide [14,15], metal oxides [16], metal sulfides [4,17,18], metal phosphates [11], and polymers [19] to fabricate HSCs. Among them, layered ternary metal sulfides are promising candidates due to their unique intrinsic properties, which include a high conductive nature, high theoretical capacity, low cost, and high specific power and energy with excellent cyclic stability due to the low electronegativity of sulfur [20,21]. In addition, the layered structure provides large interlayered spacing, which stores a large capacity using deep intercalation and limited diffusion, confirming a battery-type material [11].

Very few reports are available on Ni- and Cr-based ternary metal sulfides for SC applications. For example, Xu et al. synthesized mesoporous NiCo_2_S_4_ microspheres using a solvothermal method and obtained a specific capacity of 857 C/g at the current density of 1 A/g [22]. Similarly, Ni et al. reported a maximum specific capacity of 803.08 C/g at a current density of 1 A/g for the core-cell structure of NiMn_2_O_4_@NiMn_2_S_4_ nanoflowers@nansheets synthesized using a hydrothermal method [23]. Du et al. prepared NiMoS_4_ and nickel-copper-sulfide for HSCs and achieved maximum specific capacities of 313 and 422.37 C/g at a current density of 1 A/g, respectively [24,25]. Hai et al. reported Cr-doped (Co, Ni)_3_S_4_/Co_9_S_8_/NiS_2_ nanowires/nanoparticles using the hydrothermal method and achieved a high capacity of 1117 C/g [26]. Bulakhe et al. reported on a polyhedron-structured CuCr_2_S_4_ cathode using the hydrothermal method and obtained a capacity of 1536 C/g at 1 A/g current density [17]. Thus far, there have been no reports on NiCrS_4_ and its hybrid/composite materials, so we focused this research on this topic. The literature in Table S1 concludes that the hydrothermal method efficiently synthesizes ternary metal disulfides and their hybrid/composite materials.

We synthesized NiCr_2_S_4_ (NCS) and hybridized it using single-layered graphene oxide (GO) and prepared nanohybrid NiCr_2_S_4_/rGO (NCSG) materials. GO has been successfully applied to supercapacitors and batteries owing to its low production cost and outstanding physicochemical and electrochemical properties [27,28,29]. The conducting GO plays an important role in hybridization, and a reduced GO (rGO) amount was successfully engineered to enhance the performance of the NCSG. The optimized nanohybrid NCSG-2 provides a high specific surface area of 685.84 m^2^/g compared to bare NCS at 149.37 m^2^/g, resulting in a mesoporous nature with a pore diameter of around 15 nm. The NCSG nanohybrid material displays excellent capacity and retention with outstanding cyclic stability. An HSC device was devised using NCSG-2 as the cathode and rGO as the anode, denoted as NCSG//rGO. The HSC demonstrated outstanding specific energy and power with excellent cyclic stability. While submitting the manuscript, we found that this is the first report on an HSC study.

## 2. Experimental Section

### 2.1. Chemicals

All chemicals were purchased from Sigma-Aldrich (St. Louis, MO, USA). Nickel chloride (NiCl_2_), chromium chloride (CrCl_3_), hexamethylenetetramine (C_6_H_12_N_4_), sodium sulfide (Na_2_S), and GO ink were used prior to purification. Nickel foam (NF) with >99.99 purity was purchased from MTI Korea (Seoul, Korea).

### 2.2. Experimental Details on NiCr_2_S_4_ and NiCr_2_S_4_/rGO

**Scheme 1:**
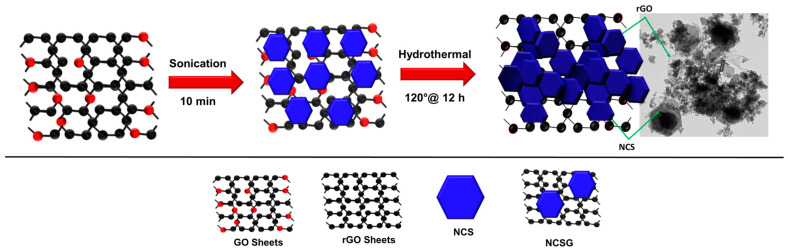
Synthesis process of Ni-Cr-S/reduced graphene oxide (NCSG).

The synthesis process for NCSG is described in Scheme 1. A chemical bath was prepared using 0.17 g of NiCl_2_, 0.38 g of CrCl_3_, and 0.22 g of Na_2_S, serving as nickel, chromium, and sulfur precursors, respectively. Additionally, 0.99 g of HMT was introduced as a complexing agent during reactions. The chemical bath was prepared using water as an aqueous solvent. The chemical bath solution was stirred for 30 min at a ramping speed of 100 rpm. The prepared homogeneous chemical bath was poured into a 100 mL Teflon liner. The Teflon liner was closed and assembled in a stainless-steel case. The autoclave was kept in a convection oven for 12 h at 120 °C. After completing the reaction, the autoclave was cooled to the atmospheric temperature. The detailed reaction mechanism is explained in Equations S1–S6 in Note S1. The obtained product was repeatedly cleaned and filtered with deionized water and ethanol. The prepared NCS powder was dried for 12 h at 60 °C. A similar experiment was repeated with the addition of a single-layered GO ink of 10%, 20%, and 30% volume ratios to prepare hybrid NCSG materials. The prepared samples were labeled NCSG-1, NCSG-2, and NCSG-3, respectively. All of the NCS and NCSG samples were annealed at 450 °C for 2 h in an inert atmosphere. All prepared samples were used for further physicochemical and electrochemical characterizations.

### 2.3. Material Characterizations

All physicochemical characterizations for NCS, NCSG-1, NCSG-2, and NCSG-3 samples were investigated. Structural characterizations were performed using X-ray powder diffraction (XRD, wavelength: 0.15406 nm) and X-ray photoelectron spectroscopy (XPS) techniques. Morphological characterizations were investigated using field-emission scanning electron microscopy (FE-SEM) and high-resolution transmission electron microscopy (HR-TEM). A porosity study was performed using the Brunauer–Emmett–Teller (BET) technique. The electrochemical studies were conducted using Bio-Logic science instruments.

### 2.4. Electrochemical Characterizations

The synthesized NCS, NCSG-1, NCSG-2, and NCSG-3 samples were investigated for electrochemical studies. Prior to the experiment, NF was cleaned with 2 M HCl to remove the oxide layer on the surface of the NF. The cleaned NF was used as a current collector. In an appropriate proportion of active materials, conductive carbon black and polyvinylidene difluoride (85%, 10%, and 5%) were mixed to prepare a homogeneous slurry. The obtained slurry was uniformly coated onto the NF, and the prepared electrodes were dried at 60 °C for 12 h. Afterward, the prepared electrodes were used as working, platinum chips, and Ag and AgCl were used as the counter and reference electrodes, respectively. The aqueous 2 M KOH electrolyte was used for the entire electrochemical performance. The cyclic voltammetry (CV), charge–discharge (CD), electrochemical impedance spectroscopy (EIS), and cyclic stability were performed for three-electrode systems. Afterward, the HSC was devised using NCSG-2 as the cathode, with rGO as the anode, a porous glass fiber separator, and an aqueous KOH electrolyte. The electrochemical cell (EC-CELL) setup was used to determine the performance regarding the CV, GCD, EIS, and cyclic stability. 

## 3. Results and Discussion

The structural analysis of the prepared NCS and NCSG samples was performed based on XRD. The two major broad and intense characteristic peaks are found at the angles of 2θ: 17.20°, 30.28°, 34.81°, 35.87°, 44.63°, 49.42°, 56.18°, 59.41°, 65.07°, and 67.75°, which correspond to the (101), (110), (202), (013), (−114), (006), (−312), (215), (017), and (008) planes, respectively, belonging to the NCS (PDF card no. 01-088-0659). It confirms the formation of crystalline monoclinic NiCr_2_S_4_. The weak peaks marked with a star symbol in the plot belong to nickel sulfide. The peaks belonging to rGO in the NCSG XRD spectra are absent, which is possibly ascribed to the small percentage of rGO, so they are masked by the diffraction signal for NCS or the destruction of the regular GO during the synthesis processes through the interaction of NCS [30].

**Figure 1:**
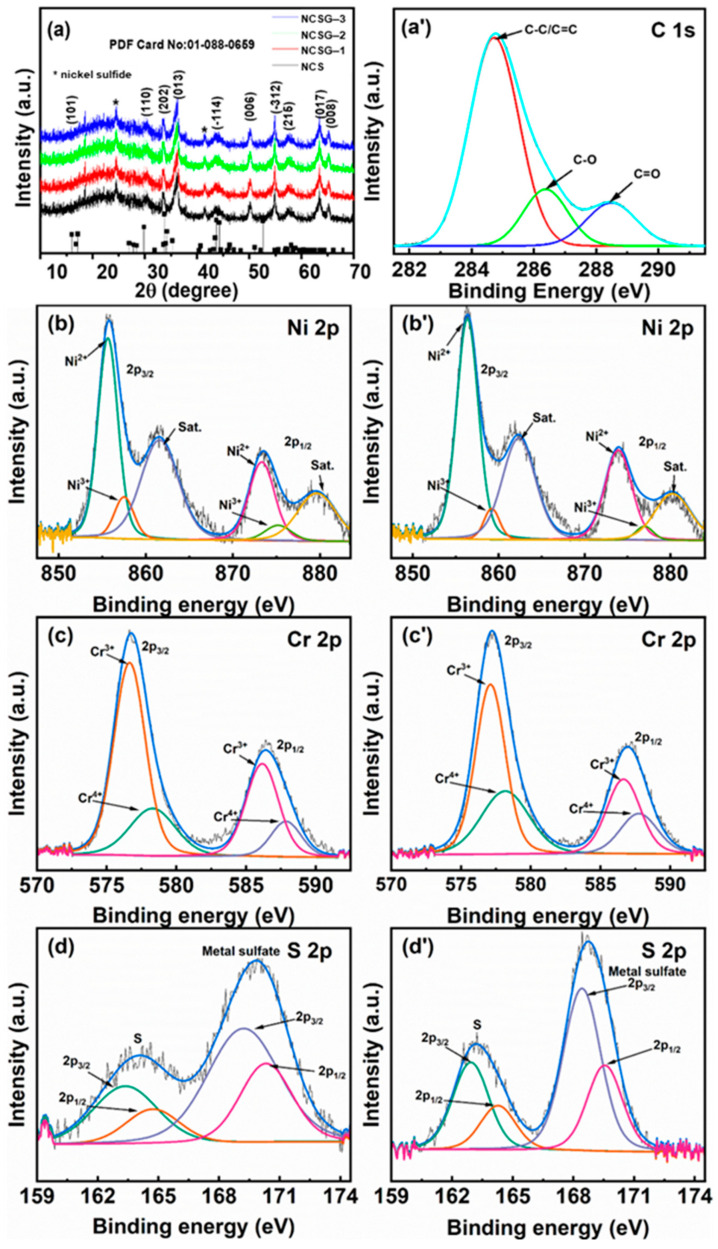
(**a**) X-ray powder diffraction spectra for NCS and all NCSG samples; high-resolution X-ray photoelectron spectroscopy spectra of (**a′**) C, (**b**,**b′**) Ni, (**c**,**c′**) Cr, and (**d**,**d′**) S elements of NCS and NCSG-2 samples, respectively.

Further, the result was confirmed with the XPS study. The elemental composition and valance states of the NCS and NCSG-2 samples were investigated using the XPS analysis. Figure S1 depicts the full survey spectrum of the NCS and NCSG-2 samples. The XPS survey confirms the coexistence of Ni, Cr, and S in NCS and the additional C element in the NCSG-2 spectrum. Figure 1a′ presents the C1s spectrum deconvoluted in three peaks at 284.7, 286.3, and 288.4 eV, belonging to the C-C/C=C, C-O, and C=O functional groups, respectively [31]. The small peaks for C-O and C=O confirm rGO formation during synthesis. The C-C/C=C functional group indicates the same carbon skeleton structure before and after the reaction; hence, rGO may provide a large specific surface area of the nanohybrid NCSG-2 material [11,32,33].

In Figure 1b,b′, the Ni 2p spectra are comparable between the NCS and NCSG-2 samples. Spin–orbit doublets of 2p_3/2_ and 2p_1/2_ are revealed; in order, Ni^2+^ peaks are located at 855.73 and 873.48 eV, Ni^3+^ at 857.33 eV and 875.1 eV, and satellite peaks at 861.83 eV and 879.68 eV, respectively [34,35,36]. The spin–orbit splitting of doublet pairs is ~17.75 eV, typical of Ni 2p doublets [35,36]. These results confirm the coexistence of Ni^2+^ and Ni^3+^ [35]. Figure 1c,c′ depict the resembling Cr 2p spectra of NCS and NCSG-2 samples. They are deconvoluted into two spin–orbit doublet pairs of 2p_3/2_ and 2p_1/2_. Cr^3+^ peaks are located at 576.73 and 586.16 eV, Cr^4+^ at 578.58 and 587.88 eV, respectively [31,37], and the spin–orbit splitting is ~9.3 eV, which is typical of Cr 2p [37], confirming the coexistence of Cr^3+^ and Cr^4+^ [26]. The S 2p spectra are depicted in Figure 1d,d′. The S 2p spectra were also deconvoluted into 2p_3/2_ and 2p_1/2_ pairs. For these pairs, S peaks are placed at 163.43 and 164.88 eV, while metal sulfate peaks are found at 169.28 and 170.38 eV, respectively [34,35,38,39,40]. The 1.15 eV spin–orbit is typical of S 2p [41]. 

**Figure 2:**
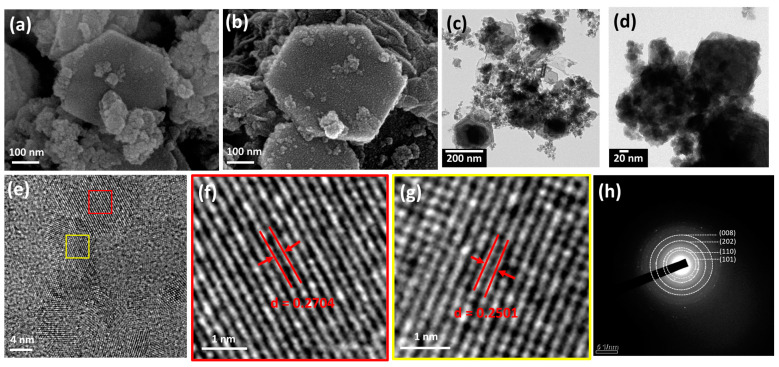
(**a**) Field-emission scanning electron microscopy images of bare NiCr_2_S_4_ (NCS), (**b**) nanohybrid NCS with 20% graphene oxide (NCSG-2), (**c**,**d**) transmission electron microscopy (TEM) images, (**e**) high-resolution TEM image, (**f**,**g**) additional high-resolution TEM images corresponding to the areas shown in Figure 2e marked in red and yellow, respectively, and (**h**) selected area electron diffraction pattern of the NCSG-2 nanohybrid sample.

The morphological study of the NCS and NCSG samples was performed using FE-SEM to determine the crystal shape, size, and morphology. The NCS sample displayed hexagonal nanosheets with a 400 nm diameter with nanoparticles in Figure 2a. Figure 2b demonstrates that the rGO sheets are covered with NCS hexagonal nanosheets with nanoparticles and form an NCSG-2 nanohybrid. Similar results for the NCSG-1 and NCSG-3 samples are depicted in Figure S2. Similar results have also been reported for nanohybrid materials in the literature [42,43]. The elemental mapping and energy-dispersive spectroscopy (EDS) spectra of the NCSG-2 sample are depicted in Figure S3. Figure S3a–e presents the uniform distribution of all individual elements (Ni, Cr, S, and C) throughout the scanned area. Furthermore, the NCSG-2 structure was examined using HR-TEM. Figure 2c,d reveals that the NCS hexagonal nanosheets and nanoparticles completely cover the rGO sheets. Figure 2e–g illustrates the high-resolution image of the nanosheets. The HR-TEM image (Figure 2e,f) depicts the parallel lattice fringes with 0.27 and 0.25 nm ‘d’-spacing values, corresponding to the (004) and (202) planes of the monoclinic structure of NCS. Figure 2h displays the selected area electron diffraction pattern and the resulting polycrystalline nature of the NCSG-2 material. Figure S4 provides the elemental mapping analysis of the NCSG-2 sample employed using the HR-TEM connected to the EDS for all constituent elements. Figure S4 suggests that the uniform distribution for Ni, Cr, S, and C is observed throughout the NCSG-2 structure.

**Figure 3:**
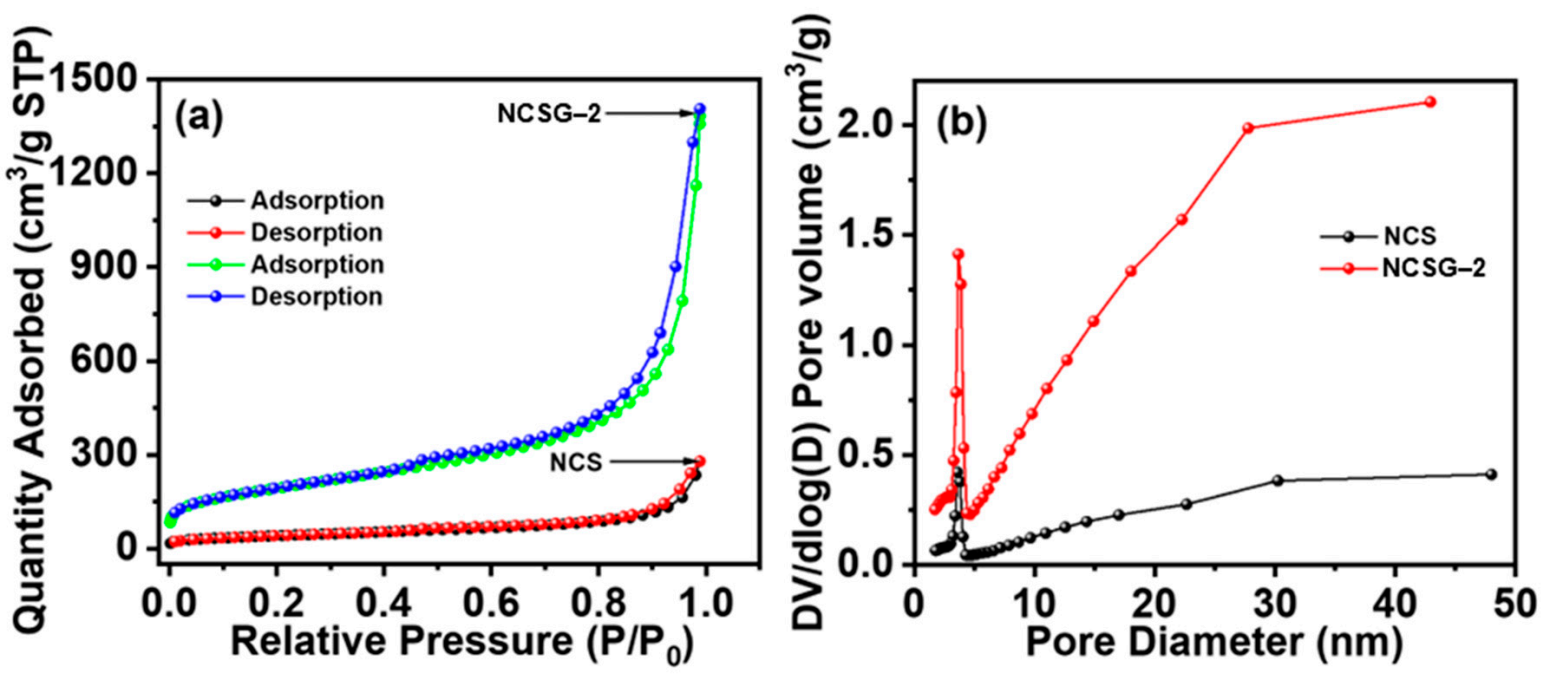
(**a**) Adsorption-desorption isotherm plot and (**b**) Barret–Joyner–Halenda (BJH) desorption pore-size distribution plot for NiCr_2_S_4_ (NCS) and NCS with 20% graphene oxide (NCSG-2).

The effects of rGO on the specific surface area, pore diameter, and pore volume of NCS were studied using the N_2_ adsorption–desorption isotherm using BET and Barret–Joyner–Halenda (BJH) measurements. The N_2_ adsorption–desorption isotherms of bare NCS and nanohybrid NCSG-2 are presented in Figure 3a. The obtained isotherms are categorized as BDDT type-IV isotherms and H_3_-type hysteresis loops, suggesting the mesoporous nature of the materials [44]. The measured specific surface areas using the BET measurements are 149.37 m^2^/g for bare NCS and 685.84 m^2^/g for NCSG-2 materials, much higher than for bare NCS. Using the BJH method, the pore-size and pore-volume distributions of bare NCS and nanohybrid NCSG-2 were investigated. The observed pore sizes of the NCS and NCSG-2 materials are 13.85 and 15.81 nm (Figure 3b), confirming a mesoporous nature. In addition, Figure 3b displayed pore volumes of 1.50 and 0.29 cm^3^/g for the NCSG-2 and NCS materials, respectively. The obtained pore sizes are <20 nm, which may reduce the electrical resistance and enhance the electrochemical activity of the materials [45]. The presence of plenty of mesopores within the nanohybrid materials facilitates the fast movement of electrolyte ions and works as ion storage, possibly enhancing electrochemical performance [45].

## 4. Electrochemical Studies

**Figure 4:**
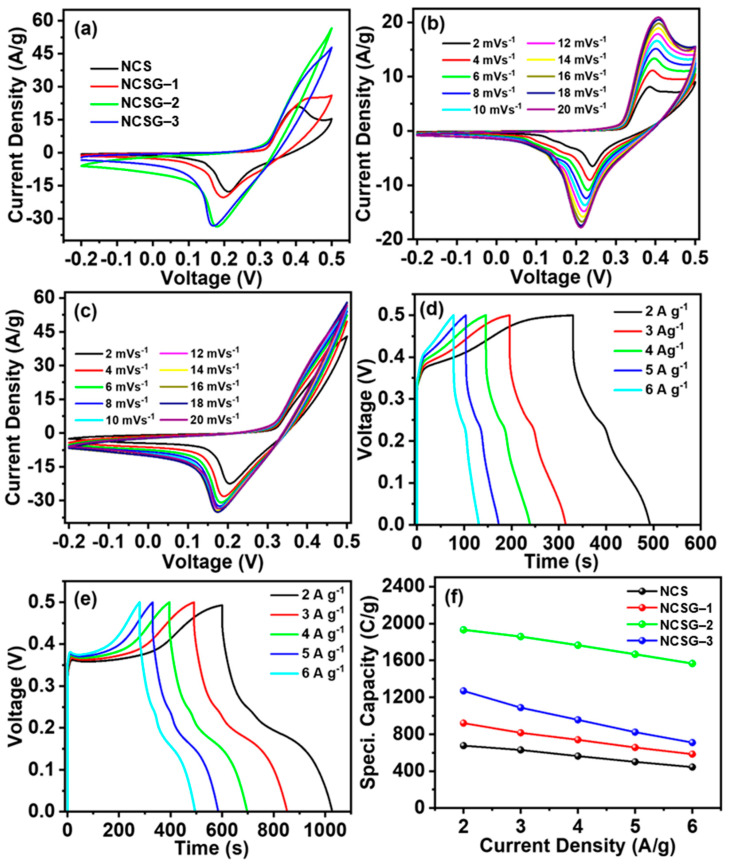
(**a**) Combined cyclic voltammetry (CV) graphs of NiCr_2_S_4_ (NCS) and NCS with 10%, 20%, and 30% graphene oxide (NCSG-1, NCSG-2, and NCSG-3) at a 20 mV/s scan rate; (**b**) CV graph of the NCS electrode (2–20 mV/s); (**c**) CV graphs of the NCSG-2 electrode (2–20 mV/s); (**d**) charge–discharge (CD) curves of the NCS electrode (2–6 A/g); (**e**) CD curves of the NCSG-2 electrode (2–6 A/g); and (**f**) graph of the current density vs. specific capacity of the NCS, NCSG-1, NCSG-2, and NCSG-3 electrodes, respectively.

The supercapacitive study was investigated using a three-electrode system consisting of NCS/NCSG electrodes serving as the working electrode and platinum chip and Ag/AgCl as the counter and reference electrodes, respectively. The aqueous 2 M KOH was used as an electrolyte. Figure 4 depicts all graphs of SC studies for NCS and NCSG electrodes. Initially, the comparative CV measurements of the bare NCS and nanohybrid NCSG-1, NCSG-2, and NCSG-3 electrodes were performed at a 20 mV/s scan rate in Figure 4a. Among them, NCSG-2 revealed an excellent capacity due to the high current value (large area under the curve). All CV curves exhibited similar shapes, including a couple of intense redox peaks. This result suggests that the specific capacity attributes, due to a fast and reversible redox reaction of Ni^2+^/Ni^3+^ and Cr^2+^/Cr^3+^, indicate battery-type faradic reactions, strongly aligning with the literature reports [14,46]. The possible redox reaction may be executed as follows:

NiCr_2_S_4_ + 4OH^−^ ⇔ NiSOH + 2CrSOH + SOH^−^ + 3e^−^ (1)

CrSOH + OH^−^⇔ CrSO + H_2_O + e^−^ (2)

NiSOH + OH^−^⇔ CrSO + H_2_O + e^−^ (3)

Figure 4b,c combined the CV curves of NCS and NCSG-2 electrodes, and Figure S5a,b depicts all CV curves for the NCSG-1 and NCSG-3 electrodes at various scan rates from 2–20 mV/s, respectively. The specific capacities of NCS and all NCSG electrodes were estimated using Equation S7. The specific capacities of NCS, NCSG-1, NCSG-2, and NCSG-3 are 764, 1109, 1960, and 1500 C/g, respectively. Battery-type faradic reactions suggest that the total stored charge contributes to diffusion-controlled (*Q_d_*) and capacitive-type (*Q_s_*) mechanisms. The following equation can be used to separate the total stored charge analysis:

*Q_t_* = *Q_s_* + *Q_d_*, (4)

where *Q_d_* = *cν*^−1/2^, *Q_t_* represents the total charge, *Q_s_* denotes the capacitive surface charge, and *c* is constant. In addition, *Q_s_* can be obtained using the graph *Q_t_* vs. *ν*^−1/2^ after extrapolating ʋ on the *y*-axis. Figure S6a plots *ν*^−1/2^ vs. the total charge for all electrodes. All calculated *Q_s_* and *Q_d_* value fragments are presented in Figure S6b for various scan rates from 2–20 mV/s for the NCS and all NCSG electrodes. The *Q_s_* values for NCS, NCSG-1, NCSG-2, and NCSG-3 electrodes are 86%, 83%, 84%, and 90%, respectively, at the 20 mV/s scan rate.

Furthermore, the charge storage ability of the NCS and NCSG electrodes was investigated using a CD study at various current density values. Figure S7a combines the curves of the CD for the NCS, NCSG-1, NCSG-2, and NCSG-3 electrodes at a 2 A/g current density. The NCSG-2 electrode displayed the maximum discharge time compared with the other electrodes, suggesting a high charge-storing ability. Similarly, Figure 4d,e and Figure S7b,c depict the CD curves of the NCS, NCSG-2, NCSG-1, and NCSG-3 electrodes at various current density values from 2–6 A/g. The specific capacities of NCS and all NCSG electrodes were estimated using the following Equation.

(5) $$Specific\;capacity\left(C/g\right)=\frac{\int^{\;}i\left(t\right)dt}{m}$$

where *i*, *t*, and *m* represent the applied current density in mA, the discharge time in seconds, and the active mass of the electrode material in mg, respectively. The specific capacity values for NCS, NCSG-1, NCSG-2, and NCSG-3 are 676, 920, 1932, and 1269 C/g, respectively. The NCSG-2 electrode displayed the highest capacity among them because the layered morphology supports a large, effective specific surface area, providing numerous active electrochemical sites.

Additionally, NCSG-2 exhibits mesoporosity (~16 nm), accelerating the diffusion of the electrolyte ions through pores and contributing to more redox reactions [12]. Based on the CD measurements, the specific capacity values for NCS and NCSG are plotted in Figure 4f. The NCSG-2 electrode delivers a much better rate capability than the NCS, NCSG-1, and NCSG-3 electrodes. The rate capabilities of the NCS, NCSG-1, NCSG-2, and NCSG-3 electrodes are 60.6%, 65.4%, 81%, and 69%, respectively. Figure 4f reveals that the bare NCS electrode had a smaller capacity than all NCSG electrodes because the conductive rGO plays a crucial role in the hybridization. Table S1 provides a comparative SC study of the Ni- and Cr-based ternary metal dichalcogenides. The specific capacity reported in the literature suggests that NCS and NCSG perform better [17,24,25].

The charge storage capabilities are directly proportional to the amount of GO hybridized in the NCSG compositions. The NCSG-2 offers a high capacity value (1932 C/g) compared to NCSG-1 (920 C/g) and NCSG-3 (1269 C/g) because the amount of GO is optimum for NCSG-2. If the GO amount is low, the probability of forming a conductive channel is low. In another case, when the amount of GO is too high, the GO sheets are likely to restack, reducing the charge storage performance [11]. 

Moreover, EIS is an important tool providing an intrinsic resistance of the active materials and the solution resistance (electrode and electrolyte interface). A comparative EIS study for NCS and NCSG electrodes was investigated. Figure S8a depicts all combined Nyquist plots for the NCS and NCSG electrodes. All hybrid NCSG electrodes displayed a semicircle in the decreasing trend compared to the bare NCS electrodes, suggesting that incorporating the GO sheets minimized the charge-transfer resistance (*R_ct_*). The low-frequency region of the Nyquist plots is ascribed to the diffusion resistance of the electrolyte (*W*). All NCSG electrodes displayed an enlarged slope, indicating that the porous structure enhanced the electrolyte ion’s kinetic transport in the hybrid electrodes. Table S2 provides the comparative values of all parameters from the fitting of the Nyquist plots for all NCS and NCSG electrodes. The NCSG-2 electrode exhibited better *R_s_*, *R_ct_*, *C_dl_*, and *W* values, respectively.

The study of the long-term stability of SCs is important for NCS and NCSG electrodes (Figure S8b). The continuous 10,000 GCD cycles were performed at a 10 A/g current density for each electrode. During the CD processes, swelling and shrinkage while intercalation and deintercalation processes result in stress and cracks within the material and interface [47]. The NCSG-1, NCSG-2, and NCSG-3 electrodes retain 90.8%, 95.2%, and 92.9%, and the NCS electrode retains 89.1% of their original values over continuous 10,000 GCD cycles. The results indicate that NCSG-2 is an excellent electrode among the studied options.

### Hybrid Supercapacitor

**Figure 5:**
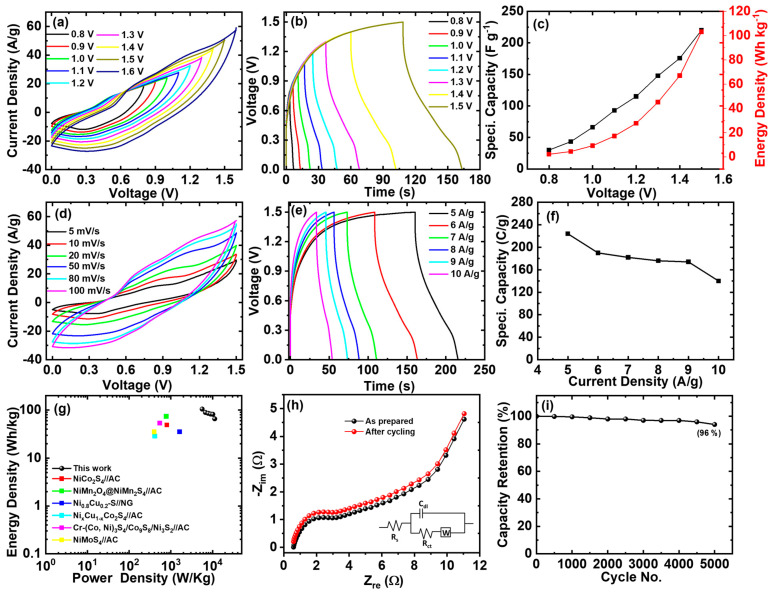
Complete hybrid supercapacitor (HSC) study: (**a**) combined cyclic voltammetry (CV) graphs from 0.8 to 1.6 V potential windows with a 100 mV/s scan rate; (**b**) combined charge–discharge (CD) curves from 0.8 to 1.6 V potential window with a 6 A/g current density; (**c**) graph of potential variation with specific capacity (black color) and energy density (red color); (**d**) CV curves from 5 to 100 mV/s of the scan rate; (**e**) CD curves from 5 to 10 A/g of the current density; (**f**) graph of the current density vs. specific capacity; (**g**) Ragone plot; (**h**) Nyquist plot; and (**i**) Capacity retention obtained from continuous 5000 CD cycles for HSC cell.

To investigate the potential of the nanohybrid NCSG-2 electrode in the full cell assembly, the HSC was devised using NCSG-2 as the positive electrode and rGO as the negative electrode. The performance of rGO (the EDLC) is studied in an aqueous electrolyte of 2 M KOH using a three-electrode assembly, and all results (CV, CD, and EIS) are provided in Figure S9. The mass balance of the anode and cathode materials was optimized using the equation provided in Note S2 to obtain the high electrochemical performance of the HSC. In the HSC, we combined two potential materials as separate electrodes to achieve a higher cell potential than individual electrodes, resulting in a high energy density. In this study, the 0 to −1 V potential for the anode is coupled with the −0.2 to 0.5 V of the cathode, providing a full cell voltage of 1.5 V (Figure S10). The CV and CD curves were examined at various potential ranges from 0 to 1.6 V to investigate the optimum operating potential range of NCSG-2//rGO HSC (Figure 5a,b) [48]. The specific capacity was calculated using CD curves for various potential ranges (Figure 5c), indicating that the capacity increases linearly with potential ranges. Similarly, a linear energy density increase was observed from 2.13 to 105 Wh/kg as the potential range increases in the range of 0.8 to 1.5 V, resulting in the effectiveness of assembling the HSC (Figure 5c).

The studies of the HSC were performed using an optimum 1.5 V potential window at various scan rates (5 to 100 mV/s). Figure 5d depicts the semi-rectangular shapes of all CV curves, suggesting a hybrid-type charge storage mechanism (EDLC and battery-type) contributed to the capacitance as a result of the positive (cathode) and negative (anode) materials. As the scan rates increased from 5 to 100 mV/s, the shapes of the CV curves continued with the same enlarged area, indicating the high performance of the HSC device. Afterward, the CD performance of the HSC device was investigated at various current density values (Figure 5e). The specific capacities were estimated using discharge curves for each current density (Figure 5f). At the lowest current density of 5 A/g, the HSC delivers 224 C/g, and for a high current density of 10 A/g, it remains at 140 C/g. The capacity retention remains at 62.5% of its original value after increasing the current density to 10 A/g. The energy and power density values of the HSC device were calculated and plotted in Figure 5g. The HSC delivers the maximum energy density of 105 Wh/kg at the power density of 5625 W/kg and the maximum power density of 11,250 W/kg at the lowest energy density of 65.62 Wh/kg. Table S3 provides a comparative study of the electrochemical performance of Ni- and Cr-based HSC devices reported earlier and presents the working NCSG-2//rGO device. Table S3 suggests that the HSC device displays superior performance compared to the reported HSC devices [23,24,25,26,35].

The electrochemical impedance measurements of the NCSG-2//rGO HSC device have been investigated from a lower frequency of 100 mHz to a higher frequency of 1 MHz (Figure 5h). The HSC delivers a smaller solution resistance (*R_s_*) and charge-transfer resistance (*R_ct_*) of 0.47 and 1.03 Ω, respectively. After completing 5000 continuous CD cycles, *R_s_* and *R_ct_* increased to 0.71 and 1.70 Ω, respectively. The enhancement in the EIS parameters is very small, suggesting the excellent performance of the electrode materials. The equivalent circuit was fitted to the Nyquist plot and depicted in the Figure 5h inset. All values of the circuit components are given in Table S4. The capacitance retention against the cycle number graph is plotted in Figure 5i. The HSC device reduced capacity retention by 4% over 5000 CD successive cycles at a 10 A/g current density. A similar study was reported for Ni- and Cr-based HSCs (Table S3). The performance of the NCSG electrodes can also be evaluated based on specific capacitance (F/g), and the converted results are shown in Figure S11 of the Supplementary Materials.

## 5. Conclusions

The mesoporous layered nanohybrid novel NCSG was successfully synthesized in a single step using a simple and inexpensive chemical route. The physicochemical and electrochemical properties of the NCSG were tuned using various amounts of single-layer GO during the nanohybrid material preparation. Mesoporous NCSG provides a four-fold greater specific surface area of 658.84 m^2^/g compared to bare NCS (149.37 m^2^/g). The conductive rGO enhanced the three-fold higher capacity of the optimized NCSG-2 (1932 C/g) compared to the bare NCS (676 C/g) materials. The NCSG-2 was used as the cathode, and rGO was used as the anode during the fabrication of the HSC. The HSC delivered a maximum specific capacity of 224 C/g at a 5 A/g density. The HSC had a maximum energy density of 105 Wh/kg and a maximum power density of 11,250 W/kg. The HSC obtained 96% cyclic stability over successive 5000 CD cycles. The robust and outstanding supercapacitive performance of the NCSG nanohybrid material suggests that it is a promising candidate for high-energy HSCs.

## Data Availability

The data presented in this study are available on request from the corresponding author.

## Supplementary Materials

The following supporting information can be downloaded at: https://www.mdpi.com/article/10.3390/ma16196598/s1, Figure S1: Entire X-ray photoelectron spectroscopy survey spectrum; Figure S2: Field-emission scanning electron microscopy images; Figure S3: Field-emission scanning electron microscopy elemental mapping; Figure S4: Energy dispersive spectroscopy mapping for each element; Figure S5: Cyclic voltammetry curves; Figure S6: Plot of the reciprocal square root of the scan rate vs. the total charge; Figure S7: Combined charge–discharge (CD) curves of NiCr_2_S_4_ (NCS) and NCS with 10%, 20%, and 30% graphene oxide; Figure S8: Nyquist plots and stability study at a 10 A/g current density for NiCr_2_S_4_ (NCS) and NCS; Figure S9: Supercapacitor study of reduced graphene oxide; Figure S10: Comparative cyclic voltammetry graphs of reduced graphene oxide (rGO; black) and NiCr_2_S_4_; Figure S11: Graph of specific capacitance vs. current density for NCS, NCSG 1, NCSG-2, and NCSG-3 electrodes; Table S1: Detailed description of Ni- and Cr-based TMD’s for supercapacitive performance using chemical methods; Table S2: Comparative data from Nyquist plot fittings; Table S3: The Ni- Cr-based HSC device study for supercapacitive parameters; Table S4: Fitting parameters. References [49,50,51,52,53] are cited in the supplementary materials.
