# Supplementary material for: Facile Synthesis of Mesoporous Nanohybrid Two-Dimensional Layered Ni-Cr-S and Reduced Graphene Oxide for High-Performance Hybrid Supercapacitors
Source: Materials (Basel). 2023 Oct 8;16(19):6598. doi: 10.3390/ma16196598 (PMC10574503; doi:10.3390/ma16196598)
Supplement: Supplementary file 1 [file materials-16-06598-s001.zip › materials-2640037-supplementary.pdf]

Supporting Information

Facile Synthesis of Mesoporous Nanohybrid Two-Dimensional Layered Ni-Cr-S and Reduced Graphene Oxide for High-Performance Hybrid Supercapacitors

Ravindra N. Bulakhe [1,2], Anh Phan Nguyen [3], Changyoung Ryu [1], Ji Man Kim [2] and Jung Bin In [1,3,*]

[1] Soft Energy Systems and Laser Applications Laboratory, School of Mechanical Engineering, Chung-Ang University, Seoul 06974, Republic of Korea; bulakhern@gmail.com (R.N.B.); asd24zx@cau.ac.kr (C.R.)
[2] Department of Chemistry, Sungkyunkwan University, Suwon 16419, Republic of Korea; jimankim@skku.edu
[3] Department of Intelligent Energy and Industry, Chung-Ang University, Seoul 06974, Republic of Korea; anhpn@cau.ac.kr
* Correspondence: jbin@cau.ac.kr

Note S1

Experimental details on $NiCr_2S_4$ and $NiCr_2S_4$/rGO

Initially, the chemical bath was prepared using the precursors of nickel chloride (0.17 g), chromium chloride (0.38 g), and sodium sulfide (0.22 g) as nickel, chromium, and sulfur sources. Hexamethylenetetramine (0.99 g) was used as a complexing agent, and water was used as a reaction solvent. The prepared chemical bath was transferred to a Teflon liner and enclosed inside a stainless steel autoclave. The autoclave was kept in a convection oven at 120°C for 12 h. After completing the reaction, the autoclave was cooled to room temperature. The precipitate was filtered and cleaned repeatedly with ethanol and water successively. The nickel-chromium-sulfide (NCS) product was dried for 12 h at 60°C. A similar reaction was repeated in addition to graphene oxide (GO) ink with different volume ratios in the chemical bath. The chemical bath was stirred for 30 min with a ramping speed of 100 rpm/s. The bath was sonicated for 15 min. The products were labeled NCSG-1, NCSG-2, and NCSG-3 for 10%, 20%, and 30% by volume of single-layered GO ink used in the above chemical bath. The possible reaction mechanism for the synthesis of $NiCr_2S_4$ is derived from the following equations:

$$Ni(Cl)_2 + C_6H_{12}N_4 \rightarrow Ni[(NH_3)_4]^{2+} + CO_2 \tag{S1}$$

$$Cr(Cl)_3 + C_6H_{12}N_4 \rightarrow Cr[(NH_3)_4]^{2+} + CO_2 \tag{S2}$$

$$(CH_2)_6N_4 + 6H_2O \rightarrow 6HCHO + 4NH_3 \tag{S3}$$

$$Na_2S \rightarrow 2Na + S^{2-} \tag{S4}$$

$$Ni[(NH_3)_4]^{2+} + S^{2-} \rightarrow NiS + 4NH_3 \quad (S5)$$

$$Cr[(NH_3)_4]^{2+} + S^{2-} \rightarrow Cr_2S_3 + 4NH_3. \quad (S6)$$

Finally, the NCS, NCSG-1, NCSG-2, and NCSG-3 were used for physicochemical and electrochemical characterizations.

Table S1: Detailed description of Ni- and Cr-based TMD's for supercapacitive performance using chemical methods.

Sr. no.	Materials	Method	Specific capacity	Stability (%@cycle)	Ref.
1	$NiCo_2S_4$	Solvothermal	857 C g^{-1}@1 A g^{-1}	80@2,000	[1]
2	$NiMn_2O_4$@$NiMn_2S_4$	Hydrothermal	803.08 C g^{-1}@1 A g^{-1}	91.5@5,000	[2]
3	$Ni_{0.8}Cu_{0.2}$-S	Sulfurization, Ion exchange reaction	422.37 C/g@1 A g^{-1}	--	[3]
4	$Ni_xCu_{1-x}Co_2S_4$	Hydrothermal	1340.48 F g^{-1}@1 A g_{-1}	80@10,000	[4]
5	Cr-(Co, $Ni)_3S_4$/ Co_9S_8/Ni_3S_2	Hydrothermal	(1117 C/g) 310.3 mAh g^{-1}@1 A g^{-1}	74.7@2,000	[5]
6	$NiMoS_4$	Chemical Coprecipitation	313 C g^{-1}@1 A g^{-1}	--	[6]
7	Cu_2WS_4	Hydrothermal	888.8 mAh g^{-1}@10 mA	--	[7]
8	C@$Cu_{0.5}Co_{2.5}S_4$	Solvothermal	1228 F g^{-1}@1 A g^{-1}	81.5@10,000	[8]
9	$NiCu_2S$	Hydrothermal	1583 F g^{-1}@ 15 mA g^{-1}	89@5,000	[9]
10	Ni-Co-Zn-S/AC	Hydrothermal	444.4 Fg-1@1 Ag-1	--	[10]
11	$CuCr_2S_4$	Hydrothermal	1536C/g@1A/g	85@10,000	[11]
12	**$NiCr_2S_4$**	**Hydrothermal**	**676 C g^{-1}@2 A g^{-1}**	**89@10,000**	**This work**
13	**$NiCr_2S_4$/rGO**	**Hydrothermal**	**1932 C g^{-1}@2 A g^{-1}**	**91@10,000**	**This work**

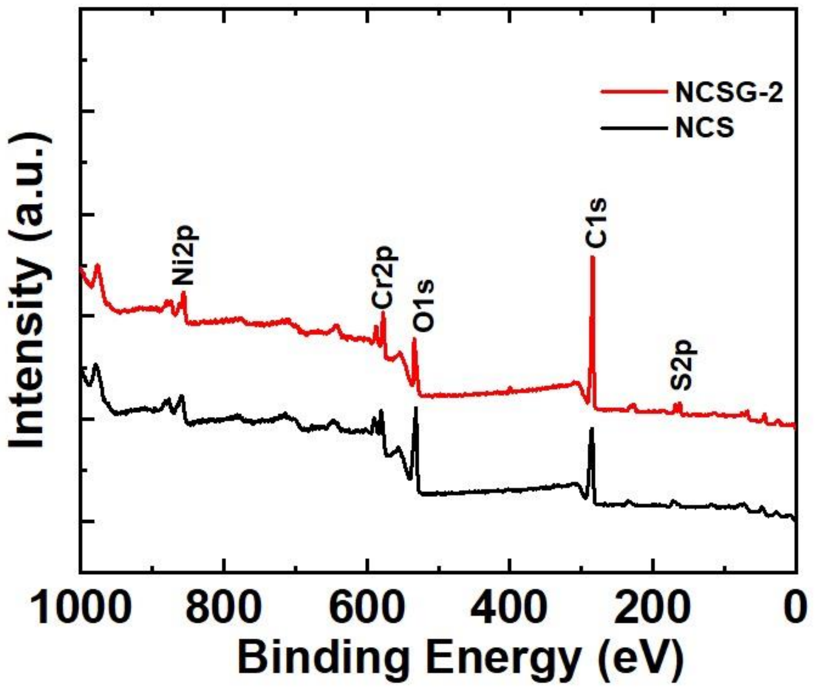

Figure S1: Entire X-ray photoelectron spectroscopy survey spectrum of $NiCr_2S_4$ (NCS) and NCS with 20% graphene oxide (NCSG-2) electrodes.

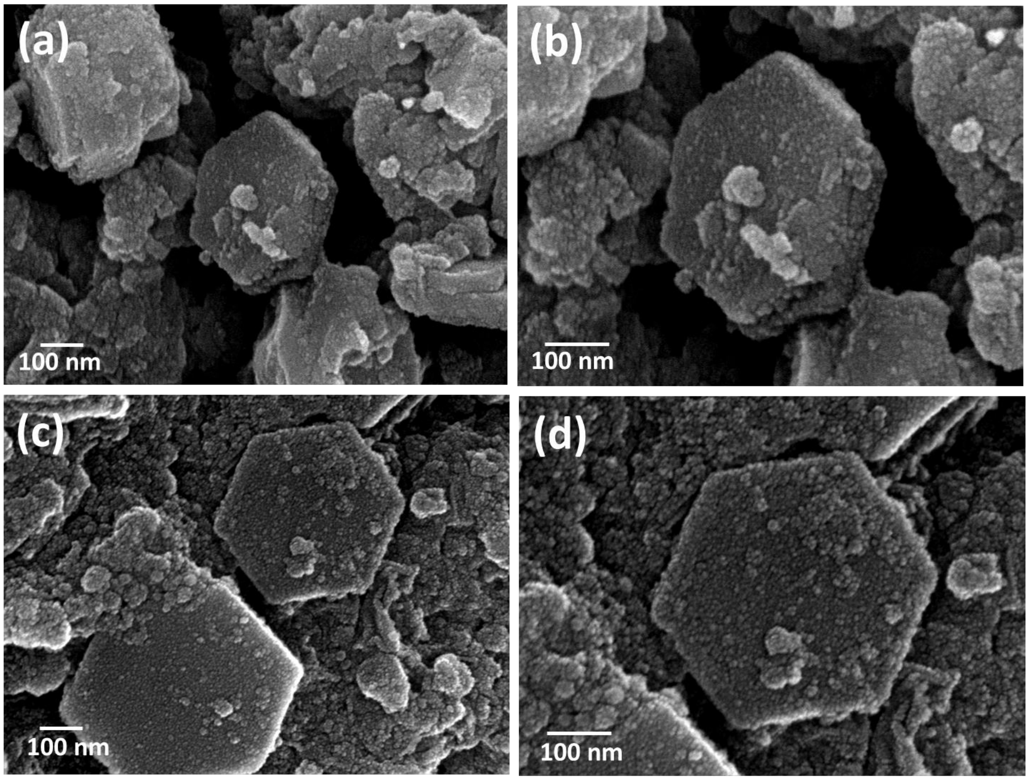

Figure S2: Field-emission scanning electron microscopy images of the (a, b) $NiCr_2S_4$ with 10% graphene oxide (NCSG-1) sample and (c, d) $NiCr_2S_4$ with 30% graphene oxide (NCSG-3) sample at various magnifications.

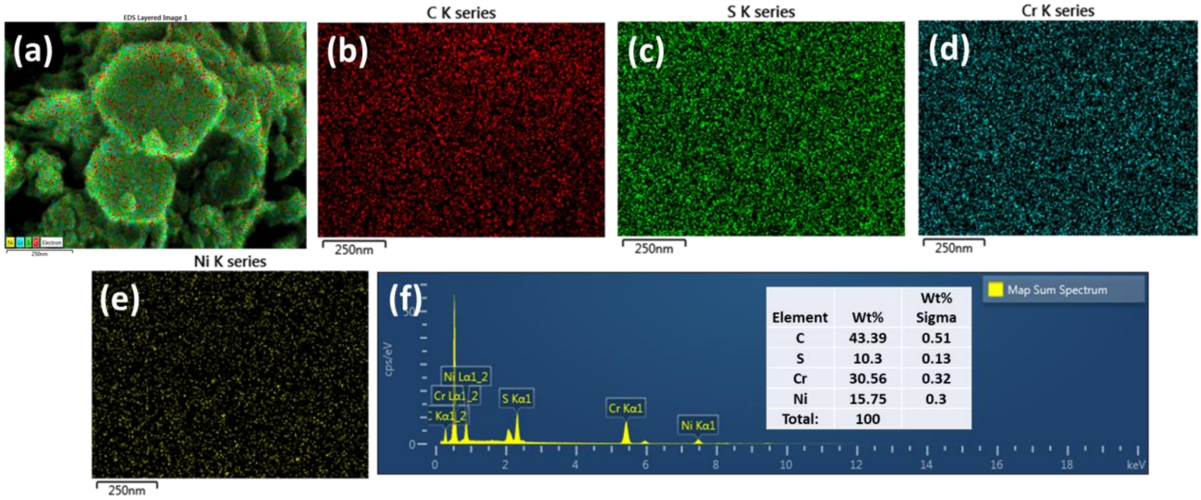

Figure S3: Field-emission scanning electron microscopy elemental mapping for each individual element: (a) combined scan of Ni, Cr, S, and C, (b) C, (c) S, (d) Cr, and (e) Ni belong to nanohybrid $NiCr_2S_4$ with 20% graphene oxide (NCSG-2) samples, and (f) EDAX spectrum; inset figure provides weights and weight percentages of the individual elements.

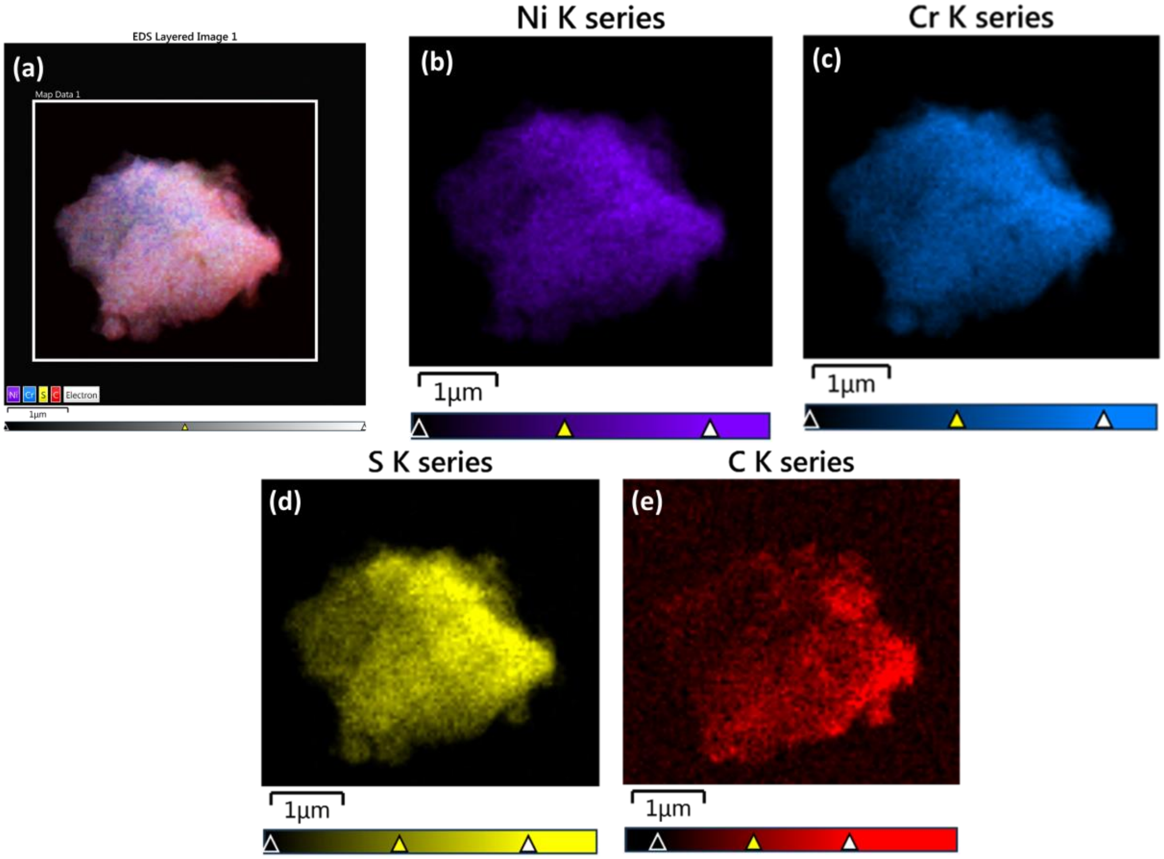

Figure S4: Energy dispersive spectroscopy mapping for each element: (a) combined scan of Ni, Cr, S, and C, (b) Ni, (c) Cr, (d) S, and (e) C belong to nanohybrid $NiCr_2S_4$ with 20% graphene oxide (NCSG-2) samples.

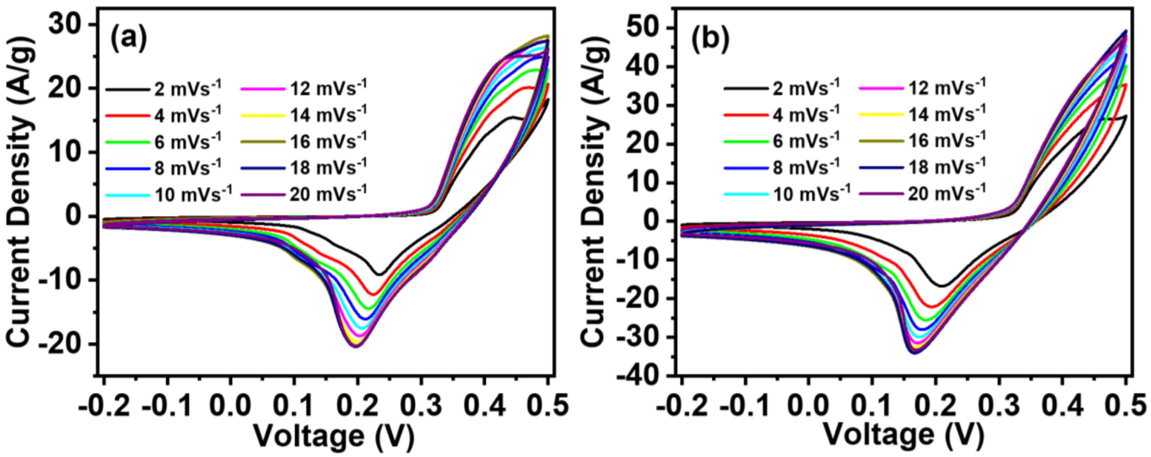

Figure S5: (a) Cyclic voltammetry curves of $NiCr_2S_4$ with 10% graphene oxide (NCSG-1) and (b) $NiCr_2S_4$ with 30% graphene oxide (NCSG-3) electrodes at various scan rates from 2 to 20 mV/s.

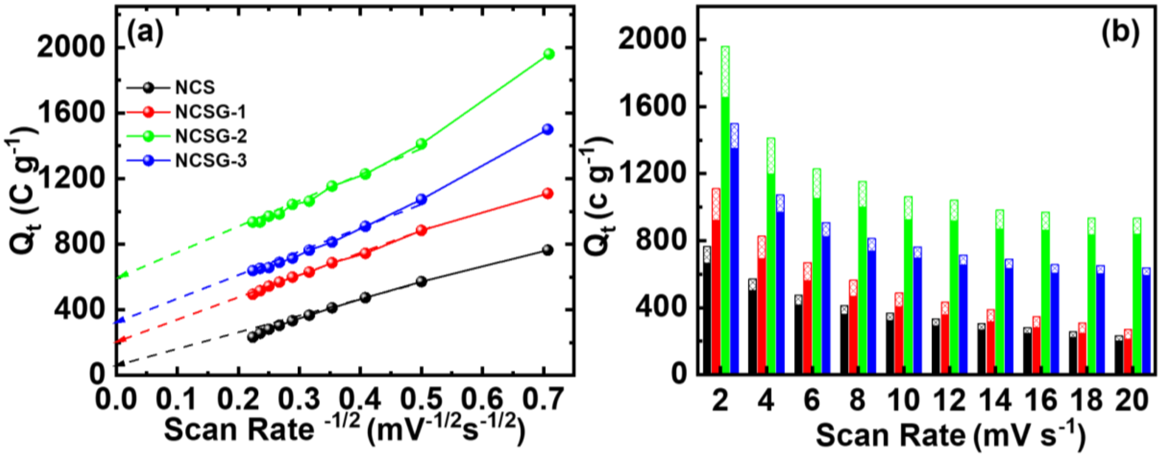

Figure S6: (a) Plot of the reciprocal square root of the scan rate vs. the total charge for $NiCr_2S_4$ (NCS) and NCS with 10%, 20%, and 30% graphene oxide (NCSG-1, NCSG-2, and NCSG-3, respectively) and (b) graph of the total stored charge contributions of surface capacitive-type (filled) and diffusion controlled-type (patterned) mechanisms for NCS (black), NCSG-1 (red), NCSG-2 (green), and NCSG-3 (blue).

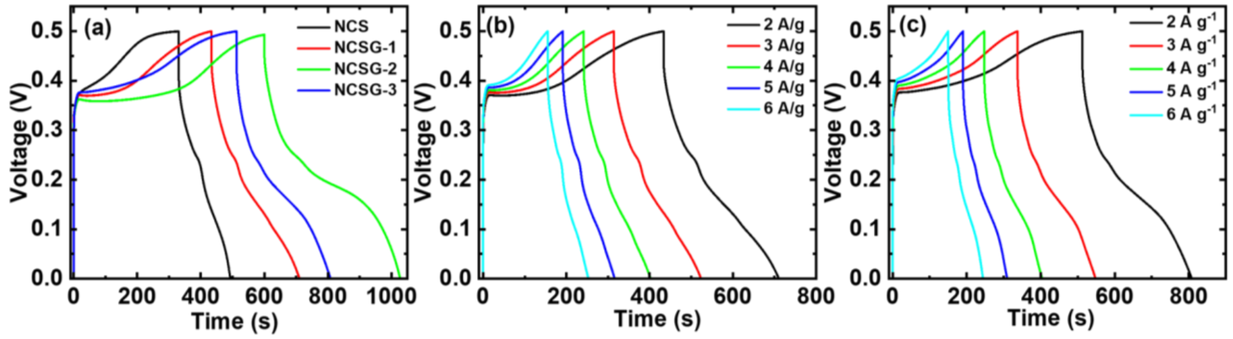

Figure S7: (a) Combined charge–discharge (CD) curves of $NiCr_2S_4$ (NCS) and NCS with 10%, 20%, and 30% graphene oxide (NCSG-1, NCSG-2, and NCSG-3, respectively) at a current density of 2 A/g, (b) CD curves of NCSG-1, and (c) CD curves of NCSG-3 electrodes at current density values from 2 to 6 A/g.

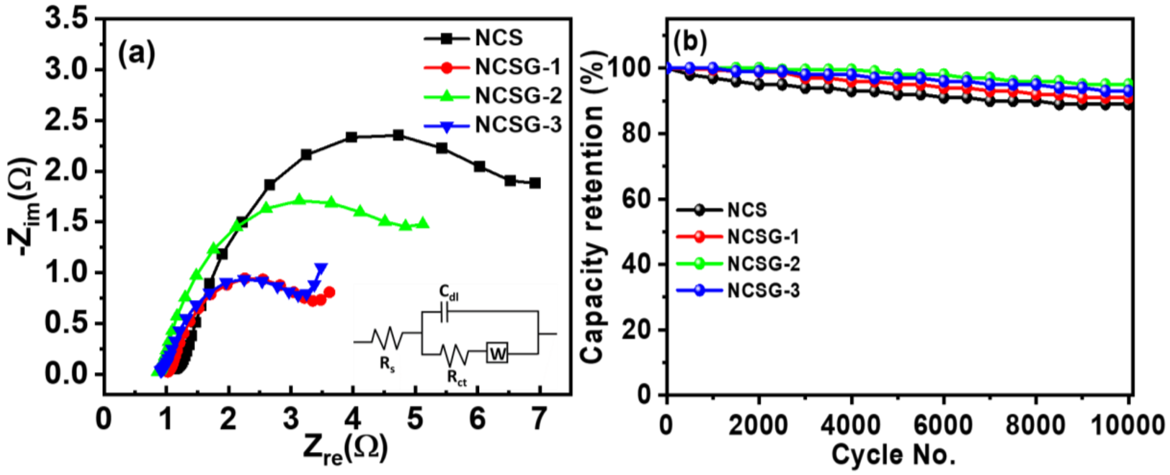

Figure S8: (a) Nyquist plots and (b) stability study at a 10 A/g current density for $NiCr_2S_4$ (NCS) and NCS with 10%, 20%, and 30% graphene oxide (NCSG-1, NCSG-2, and NCSG-3, respectively) electrodes.

Table S2: Comparative data for all $NiCr_2S_4$ (NCS) and NCS with 10%, 20%, and 30% graphene oxide (NCSG-1, NCSG-2, and NCSG-3, respectively) electrodes from Nyquist plot fittings.

Sr. no.	R_s (Ω)	R_{ct} (Ω)	C_{dl} (mF)	W (Ω $s^{-1/2}$)
**NCS**	1.13	1.20	0.61	6.92
**NCSG-1**	1.02	0.83	0.38	3.62
**NCSG-2**	0.86	0.18	0.16	5.11
**NCSG-3**	0.90	0.58	0.23	3.48

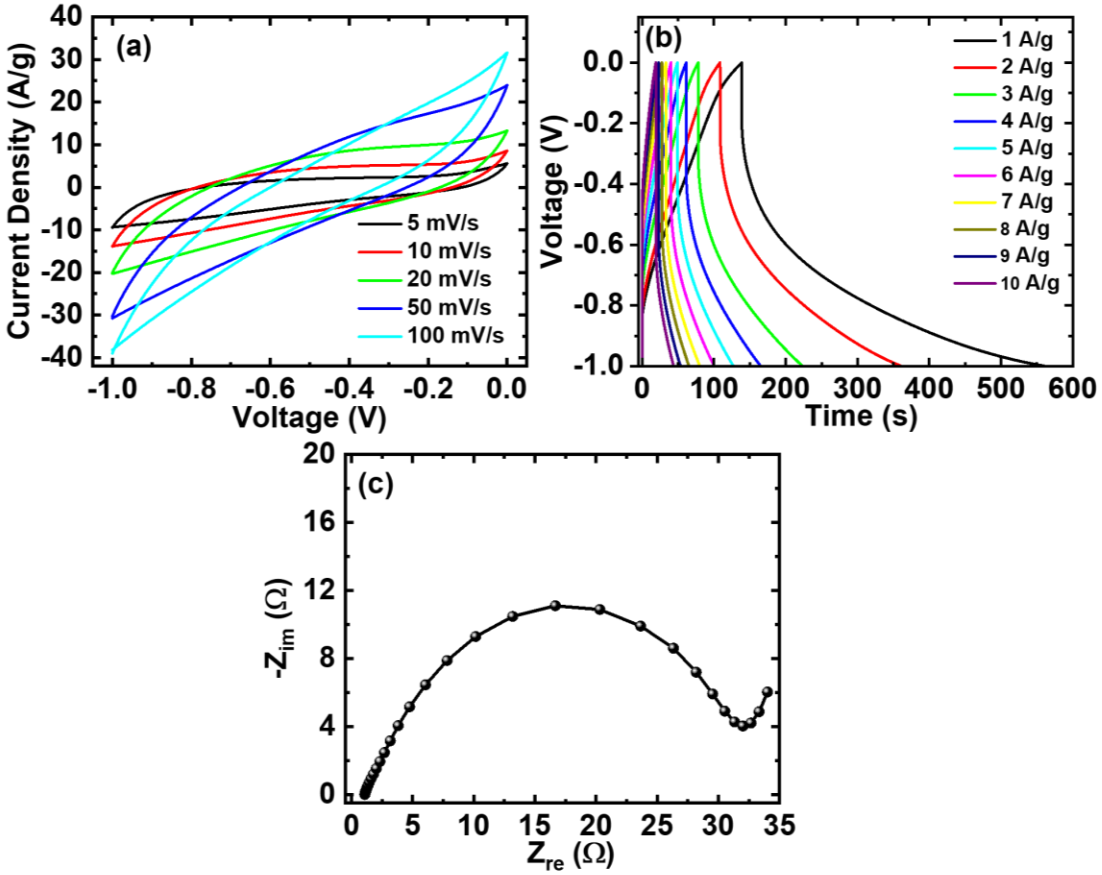

Figure S9: Supercapacitor study of reduced graphene oxide: (a) Cyclic voltammetry curves at various scan rates from 5 to 100 mV/s, (b) charge–discharge curves at various current density values from 1 to 10 A/g, and (c) the Nyquist plot.

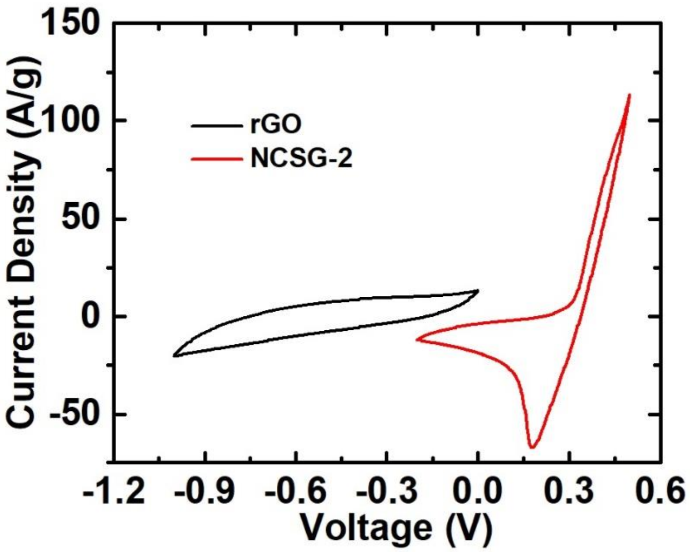

Figure S10: Comparative cyclic voltammetry graphs of reduced graphene oxide (rGO; black) and $NiCr_2S_4$ with 20% GO (NCSG-2; red) electrodes at the scan rate of 20 mV/s.

Table S3: The Ni- Cr-based HSC device study for supercapacitive parameters.

**Sr. no.**	**HSC cell**	**Spec. capac. @ current density (F/g@A/g)**	**Energy density (Wh/Kg)**	**Power density (W/Kg)**	**Stability (%@cycle)**	**Ref.**
1	$NiCo_2S_4$//AC	130.8 @ 0.5	48.7	801	81@10,000	[1]
2	$NiMn_2O_4$@$NiMn_2S_4$//AC	171.6 @ 1	73.65	775.04	--	[2]
3	$Ni_{0.8}Cu_{0.2}$-S//NG	95.9 @ 2	35.3	1627	69@10,000	[3]
4	$Ni_xCu_{1-x}Co_2S_4$//AC	105.8 @ 0.5	28.8	412.5	71.7@10,000	[4]
15	Cr-(Co, Ni)$_3S_4$/ Co_9S_8/Ni_3S_2//AC	44.7 @ 1	53.24	543.26	64@10,000	[5]
6	$NiMoS_4$//AC	101.3 @ 0.5	35	400	82@10,000	[6]
7	CWS//Graphene	107.93@1	48.57	321.42	92@10000	[7]
8	**NCSG-2//rGO**	**224 C/g @ 5 A/g**	**105**	**5625**	**96@5,000**	**This work**

Table S4:

**Parameter**	R_s **(Ω)**	R_{ct} **(Ω)**	C_{dl} **(mF)**	W **(Ω $s^{-1/2}$)**
**As prepared**	0.47	1.03	0.80	10.81
**After cycling**	0.71	1.70	0.93	12.56

Note S2

Electrochemical calculations were performed using the following formulas:

Measurement of the specific capacity from the cyclic voltammetry curves:

$$Specific\ capacity\ (C/g) = \frac{\int i(v)dV(AV)}{m(g)v\left(\frac{V}{s}\right)}, \quad \text{(S7)}$$

where the integration provides the total volumetric charges in ampere volts, v denotes the scan rate, and m represents the mass loading.

Evaluation of the specific energy and specific power from the GCD curves [12]:

$$Energy\ density\ (E_g, Wh/kg) = \frac{1}{M}\int_0^{td} iVdt \quad \text{(S8)}$$

$$Power\ density\ (W/kg) = \frac{E_g}{t_d}, \quad \text{(S9)}$$

where t_d denotes the discharge time.

Mass balancing for both electrodes to assemble the hybrid supercapacitor:

$$\frac{m_+}{m_-} = \frac{C_-\,V_-}{C_+V_+}, \quad \text{(S10)}$$

where m_+ and m_-, C_+ and C_-, and V_+ and V_- denote the mass (g/cm^2), capacitance, and potential range values for the positive and negative electrodes, respectively.

Note S3

Capacitance Evaluation

The specific capacitance is calculated using the following formula.

$$Specific\ capacitance\ (F/g) = \frac{\int i(t)dt}{\Delta V\, m} \quad \text{(S11)}$$

where, i represents the applied current density in mA, t represents the discharge time in second, ΔV represents potential window, and m represents the active mass of the materials in mg, respectively.

The capacity data shown in Figure 4f, 5c, and 5f are converted to capacitance data (F/g) based on the above calculations and shown in Figure S11a, b, and c, respectively, below.

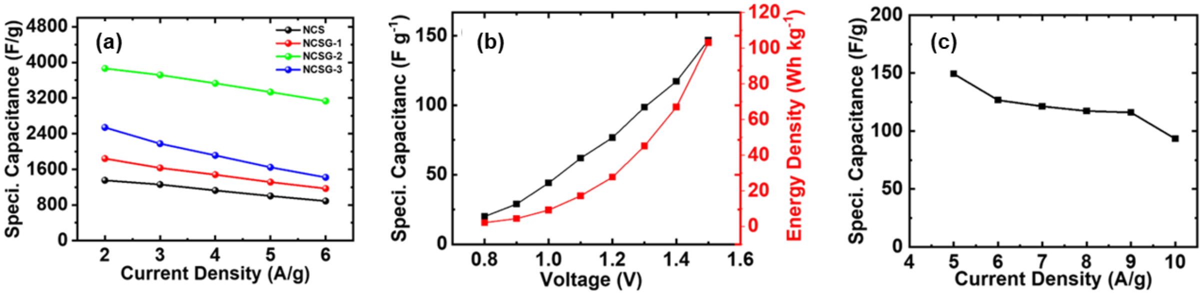

Figure S11: (a) Graph of specific capacitance vs. current density for NCS, NCSG-1, NCSG-2, and NCSG-3 electrodes, (b) graph of specific capacitance (black color) and energy density (red color) variations with potential change for the hybrid supercapacitor, (c) graph of specific capacity vs. current density for the hybrid supercapacitor.

References

[1] H. Xu, P. Chen, Y. Zhu, Y. Bao, J. Ma, X. Zhao, Y. Chen, Self-assembly and controllable synthesis of high-rate porous NiCo2S4 electrode materials for asymmetric supercapacitors, Journal of Electroanalytical Chemistry, (2022) 116688.
[2] X. Lv, X. Min, L. Feng, X. Lin, Y. Ni, A novel NiMn2O4@ NiMn2S4 core-shell nanoflower@ nanosheet as a high-performance electrode material for battery-type capacitors, Electrochimica Acta, 415 (2022) 140204.
[3] D. Du, R. Lan, J. Humphreys, H. Amari, S. Tao, Preparation of nanoporous nickelcopper sulfide on carbon cloth for high-performance hybrid supercapacitors, Electrochimica Acta, 273 (2018) 170-180.
[4] W. Chen, P. Yuan, S. Guo, S. Gao, J. Wang, M. Li, F. Liu, J. Wang, J. Cheng, Formation of mixed metal sulfides of NixCu1− xCo2S4 for high-performance supercapacitors, Journal of Electroanalytical Chemistry, 836 (2019) 134-142.
[5] Y. Hai, K. Tao, H. Dan, L. Liu, Y. Gong, Cr-doped (Co, Ni) 3S4/Co9S8/Ni3S2 nanowires/nanoparticles grown on Ni foam for hybrid supercapacitor, Journal of Alloys and Compounds, 835 (2020) 155254.
[6] D. Du, R. Lan, J. Humphreys, W. Xu, K. Xie, H. Wang, S. Tao, Synthesis of NiMoS4 for high-performance hybrid supercapacitors, Journal of The Electrochemical Society, 164 (2017) A2881.

[7] P. Pazhamalai, K. Krishnamoorthy, S. Sahoo, V.K. Mariappan, S.J. Kim, Copper tungsten sulfide anchored on Ni-foam as a high-performance binder free negative electrode for asymmetric supercapacitor, Chemical Engineering Journal, 359 (2019) 409-418.
[8] Z. Wang, Z. Zhu, Q. Zhang, M. Zhai, J. Gao, C. Chen, B. Yang, Fabrication of N-doped carbon coated spinel copper cobalt sulfide hollow spheres to realize the improvement of electrochemical performance for supercapacitors, Ceramics International, 45 (2019) 21286-21292.
[9] N. Kandhasamy, L.K. Preethi, D. Mani, L. Walczak, T. Mathews, R. Venkatachalam, RGO nanosheet wrapped β-phase NiCu2S nanorods for advanced supercapacitor applications, Environmental Science and Pollution Research, (2022).
[10] S. Sathish, R. Navamathavan*, Electrochemical Investigation of Ni-Co-Zn-S/AC Nano Composite for High-Performance Energy Storage Applications, ECS Journal of Solid State Science and Technology, 11 (2022) 101010.
[11] R.N. Bulakhe, C. Ryu, J.L. Gunjakar, J.B. In, Chemical route to the synthesis of novel ternary CuCr2S4 cathodes for asymmetric supercapacitors, Journal of Energy Storage, 56 (2022) 106175.
[12] T.S. Mathis, N. Kurra, X. Wang, D. Pinto, P. Simon, Y. Gogotsi, Energy Storage Data Reporting in Perspective—Guidelines for Interpreting the Performance of Electrochemical Energy Storage Systems, Advanced Energy Materials, 9 (2019) 1902007.
